# Editorial: Women in avian physiology: 2023

**DOI:** 10.3389/fphys.2024.1392506

**Published:** 2024-03-07

**Authors:** Sandra G. Velleman, Francesca Soglia, Servet Yalcin

**Affiliations:** ^1^ Department of Animal Sciences, The Ohio State University, Wooster, OH, United States; ^2^ Department of Agricultural and Food Sciences, University of Bologna, Bologna, Italy; ^3^ Department of Animal Science, Ege University, Bornova, Türkiye

**Keywords:** avian physiology, nutrition, reproduction, muscle, temperature

## Introduction

The “Women in Avian Physiology: 2023” is the second in a series of Research Topics focused on Women by Frontiers in Avian Physiology. The series on Women in Avian Physiology is aimed at showcasing research especially from early career women to help promote their careers in the area of avian physiology. Submissions from all areas of Avian Physiology were accepted. A total of 12 submissions were accepted for publication spanning the areas of Nutrition, Reproduction, Breast Muscle Physiology and Myopathies, and the Effects of Temperature.

## Nutrition

Three contributions addressed nutrition by Ashayerizadeh et al., Dayan et al., and Johnson et al. Ashayerizadeh et al. found that adding turmeric powder to diets increased growth in Japanese quail and the effect was enhanced with black pepper powder and may be an alternative to the addition of antibiotics in feed. Dayan et al. focused on improving immediate post hatch energy and breast muscle development by in-ovo feeding with creatine monohydrate. They found increased expression of genes related to muscle growth potential and an increased number of myofibers in the breast muscle. Johnson et al. reported findings with the addition of a microencapsulate feed additive containing botanicals and organic acids on jejunum and ileum health in 15-day old broiler chicks. The research showed that the microencapsulated feed additive resulted in a more anti-inflammatory phenotype in the jejunum and the ileum had a greater immunometabolic response.

## Reproduction

The area of reproduction was covered by four contributions. Brady et al. investigated how artificial insemination affects sperm storage tubules in turkey hens. The storage of sperm in the sperm tubules directly impacts hen fertility. Transcriptome analysis revealed that the inseminated group had the greatest change in the sperm storage tubule transcriptome which may have a direct effect on fertility. Kosonsiriluk et al. also studied the effects of turkey hen artificial insemination. They investigated the effect of insemination on the transcriptome of the uterovaginal junction where the semen storage tubules are located. In brief, they found that repeated inseminations caused a local immune response and increased aging of the turkey hen uterovaginal junction. Long non-coding RNA (lnc RNA) are a class of non-coding RNAs over 200 nucleotides in length and may regulate ovarian development. In Taihe Black-Bone Chickens, Huang et al. identified 136 differentially expressed lncRNAs. Network analysis of lnc-RNA-mRNA interactions identified 16 pairs of lncRNA-target gene associations with 7 differentially expressed lncRNAs with 14 target genes associated with reproductive traits. Interestingly, the target genes identified were primarily associated with follicle and ovary development. Keel bone damage in Japanese Quail hens was addressed by Hilebrand et al. using a radiography approach to detail the development of the keel bone. Damage to the keel bone is an animal welfare issue in laying hens and can have an occurrence up to 100% within a single flock. They found between 8 and 19 weeks of age that there was decreased radiographic density, lateral surface area and length of the keel bone. Furthermore by 23 weeks of age, 82% of the quail hens had deviations of the keel bone.

## Breast muscle physiology and myopathies

The broiler breast muscle is the most economically valuable cut due to increasing consumer demand. To meet this increase in consumer demand, the poultry industry has developed broiler lines with improved growth rates, increased meat yield, and higher feed efficiencies. Despite the improvements, breast muscle myopathies like Wooden Breast have emerged in recent years. Alnahhas et al. provided a comprehensive review of hypoxia-inducible factor 1 and how it plays a key role in the development of these conditions. Hypoxia is one of the primary causes of broiler breast muscle myopathies. The review included a discussion of the causes and consequences of hypoxia with focus given to the hypoxia-inducible factor pathway. In an opinion paper by Xu and Velleman on the role of mTOR pathway in breast muscle growth and development, the importance of mTOR signal transduction in regulating muscle fiber hypertrophy and satellite cell mediated growth was discussed. They viewed the mTOR pathway as being critical in breast muscle growth through its stimulation of myofiber protein synthesis and regulating satellite cell-mediated myogenesis. Satellite cells are located at the periphery of each muscle fiber and are responsible for all post hatch muscle fiber growth and the regeneration of muscle.

## Effects of temperature

Uyanga et al. did a bibliometric analysis of papers published in the area of poultry research from 2000 to 2021. They found that the top 10 globally cited manuscripts focused on the effects heat stress, alleviating heat stress, and the relationship between oxidative stress and heat stress poultry in poultry. The findings of this literature search underscore the concern associated with climate change and its association with heat stress. The sensitivity of primary broiler pectoralis major muscle satellite cells to temperature was investigated by Gregg et al. Since broiler body temperature is 41°C and *in vitro* assays of satellite cells are typically run at 38°C, experiments were run to determine the effect of temperature on broiler satellite cell myogenesis. It was found that culturing at 41°C compared to 38°C altered satellite cells myogenic kinetics with promoting a more rapid progression through the myogenic process and increase the number of apoptotic cells. Thus, birds that are thermally stressed post hatch may have altered myogenesis and post hatch muscle growth.

